# Removal of oxygen demand and nitrogen using different particle-sizes of anthracite coated with nine kinds of LDHs for wastewater treatment

**DOI:** 10.1038/srep15146

**Published:** 2015-10-12

**Authors:** Xiangling Zhang, Lu Guo, Yafen Wang, Congying Ruan

**Affiliations:** 1 School of Civil Engineering and Architecture, Wuhan University of Technology, Wuhan 430070, China; 2 School of Environmental Studies, China University of Geosciences, Wuhan 430074, China

## Abstract

This paper reports the application of anthracite particles of different sizes and coated with nine kinds of layered double hydroxides (LDHs) varying in M^II^–M^III^ cations, as alternative substrates in the simulated vertical-flow constructed wetland columns. Effects of LDHs-coating and particle size of modified anthracites were examined to evaluate their abilities in removing oxygen demand and nitrogen from sewage wastewater. Results showed that LDHs modification effectively enhanced the removal of nitrogen and organics. The removal efficiencies of total nitrogen (TN) , ammonia and chemical oxygen demand (COD) were best improved by 28.5%, 11.9% and 4.1% for the medium particle size (1–3 mm), followed by 9.2%, 5.5% and 13.6% for the large size (3–5 mm), respectively. Only TN removal was improved up to 16.6% for the small particle size (0.5–1 mm). Nitrate tended to accumulate and fluctuate greatly across all the treatments, probably due to the dominancy of aerobic condition in the vertical-flow columns. Overall, MgFe-LDHs was selected as the best-modified coating for anthracite. The results suggested LDHs modification would be one of the promising strategies to provide new-types of highly efficient and lasting wetland substrates.

The application of constructed wetlands (CWs) has greatly expanded in recent decades in China and worldwide, as a promising technology for wastewater treatment^1^,^2^,^3^. Wetland substrates play an important role in removing various pollutants through physical filtration, chemical adsorption and biofilm formation^4^,^5^,^6^. The type, gradation, combination and modification of wetland substrates could all directly affect the treatment performance of CWs^7^,^8^. Although there are a variety of natural wetland substrate materials, including anthracite, zeolite, bio-ceramic, vermiculite, slag and other fillers^8^,^9^,^10^, they tend to be saturated with decreasing removal efficiency due to limited adsorption capacity.

Anionic layered double hydroxide (LDHs) hydrotalcite-like minerals, represented by hydrotalcite, are comprised of paralleled and positively charged layers with intercalated anions and water molecules^11^. Owing to its exchangeable interlayer anions, structural collapses memory effect, self-assemble main laminates and other features, LDHs have been widely used in the fields of polymer composite materials, catalytic materials, environmental control and etc.^12^,^13^,^14^,^15^. A number of studies have focused on the adsorption property and capacity of LDHs for the removal of phosphate^16^,^17^,^18^, chromium (Cr_2_O_7_^2−^)^19^, selenium (SeO_3_^2-^ and SeO_4_^2−^)^20^, as well as the virus and bacteria^21^,^22^. Instant synthesis of LDHs showed good purification effects for polluted industrial wastewater, such as printing and dyeing wastewater and high fluorine water^23^,^24^. However, the application of powdered pure LDHs in CWs appears to be an appreciable problem still unsolved due to the small particle size and the low density, and the application of LDHs as filtering substrate in wastewater treatment systems is still lacking.

Our previous studies showed that natural anthracite achieved the best removal performance for phosphorus and organics among eight different filter media^10^. Anthracite was further screen out as an optimal substrate for LDHs-modification compared to biological ceramsite and zeolite, in which only one MgFe-LDHs coating was tested^25^. It was shown that different kinds of metal cations, i.e., Zn^2+^, Co^2+^ and Mn^2+^, influenced the microbial activity and polysaccharide content of natural substrate (unmodified) biofilms in different ways^26^. We assumed that the modified substrates with different LDHs-coatings varying in di- and trivalent metal cations would achieve different improved effects on treatment performance of CWs. However, such information is largely unknown.

In this study, the modified anthracites with nine kinds of LDH-coatings varying in M^II^-M^III^ cations were used as wetland substrates in the vertical-flow constructed wetland columns treating sewage wastewater. Modified anthracites of different sizes (large, medium and small) were also tested. This study aimed to (i) evaluate the performance of different modified substrates for removal of oxygen demand and nitrogen; (ii) screen out the best combinations of metal compounds and particle size for preparation of LDHs-modified anthracite.

## Materials and Methods

### Preparation of the LDHs-modified substrates

Raw anthracites were purchased from Zhengzhou, China and sieved into three different particle sizes, i.e., a large size (3–5 mm), a medium size (1–3 mm) and a small size (0.5–1 mm). The BET surface area of the original substrate was 0.5642 m^2^·g^−1^ for the small size, 0.6644 m^2^·g^−1^ for the medium size and 0.2366 m^2^·g^−1^ for the large size, respectively, determined on a Micromeritics ASAP2020 analyzer. Nine LDHs with varying M^II^-M^III^ cations including Ca-Fe, Zn-Fe, Mg-Fe, Ca-Co, Zn-Co, Mg-Co, Ca-Al, Zn-Al and Mg-Al were prepared in 2:1 molar ratios from their respective chloride salts (AR grade). The above LDHs-coatings were prepared by the co-precipitation at constant pH method, by adding simultaneously two solutions, 0.2 mol·L^−1^ of M^II^Cl_2_ and 0.1 mol·L^−1^ of M^III^Cl_3_ into a 2 L beaker containing raw anthracites washed with distilled water and heated to 80 °C in a water bath. The volume of each metal chloride solution was around 20 mL, depending on the precipitation amounts of different metal combinations. The pH of the solution was monitored in real-time and 10% NaOH solution was added dropwise to the beaker to help stabilize the pH value around 11–12. After vigorously stirring for 4 hr, the solid substrates were taken out, centrifuged at 1000–1500 rpm for 10 min and washed till the pH reaching 7 (neutral). Finally, the modified anthracites were obtained with LDHs-coating film after oven drying for 16 hr. There were totally 27 modified anthracite substrates obtained for the experimentation, including nine kinds of LDHs-coatings for each of the three particle sizes.

### Morphological characterization of the substrates

The morphological characteristics of the original and modified substrates were examined by a Zeiss Ultra Plus field-emission scanning electron microscope (FESEM), using an accelerating voltage of 3 kV and the in-lens secondary electron detector, in Material Research and Testing Center, Wuhan University of Technology.

### Experiment set-up for sewage purification experiment

The sewage wastewater was prepared by mixing the raw influent from Long Wang Zui Sewage Treatment Plant and waters of South Lake at Wuhan, China. The average concentrations of chemical oxygen demand (COD_Cr_), total nitrogen (TN), ammonia (NH_4_^+^-N), nitrate (NO_3_^−^-N) and total phosphorus (TP) in the influent were 119.9 ± 33.7, 19.0 ± 8.5, 15.1 ± 2.9, 1.3 ± 0.6 and 2.1 ± 0.7 mg·L^−1^, respectively.

A total of 10 PVC columns (8 cm in diameter, 40 cm in height) were set-up to simulate the vertical-flow constructed wetland for the sewage purification experiment. Each column was filled with 35 cm modified anthracite substrates at the bottom, with the top 5 cm reserved. Plants were not included in the columns to simplify the system and focus on the role of the modified substrates for wastewater treatment. The influent was fed from the top of the column, filtered through the modified substrates and outflowed from the bottom, with a hydraulic load of 250 L m^−2^ d^−1^ and hydraulic retention time (HRT) of 12 hr. Before the start of the experiment, the columns were fed in batches for 10 d to let the system stabilize. The whole experiment lasted for seven months from February to August 2013, including preparation of the column systems, the modified substrates and the mixed sewage wastewater for about one month, and three experimental cycles with each granulometric class lasting two months. For each granulometric class, at least five batches were carried out, and it took five days to complete the water sampling and analyzing for each batch. The FESEM observations of all the tested substrates were also carried out prior to each experimental cycle.

### Water quality analysis

COD_cr_ was measured by potassium dichromate method. Total nitrogen (TN) was determined by UV spectrophotometry after alkaline potassium persulfate digestion, while NH_4_^+^-N and NO_3_^−^-N were determined using Nessler’s reagent colorimetric method and phenol disulfonic acid spectrophotometry, respectively^27^.

### Statistical analysis

Two-way ANOVA was used to test the differences in removal efficiencies of NH_4_^+^-N, TN, NO_3_^−^-N and COD among the three particle sizes and nine LDHs-coatings by SPSS 12.0 software (SPSS Inc., Chicago, IL, USA). One-way ANOVA, followed by a post-hoc test (Tukey HSD) for multiple comparisons, was carried out for each particle size, if the interaction effect of particle size and LDHs-coating was significant at *P* ≤ 0.05. Principal component analysis (PCA) was used for comprehensive evaluation of the treatment performance of the tested substrates, based on the data of average removal efficiencies of NH_4_^+^-N, TN, NO_3_^−^-N and COD, by PAST (PAlaeontological Statistics).

## Results and Discussion

### Morphological characteristics

**Figure 1:**
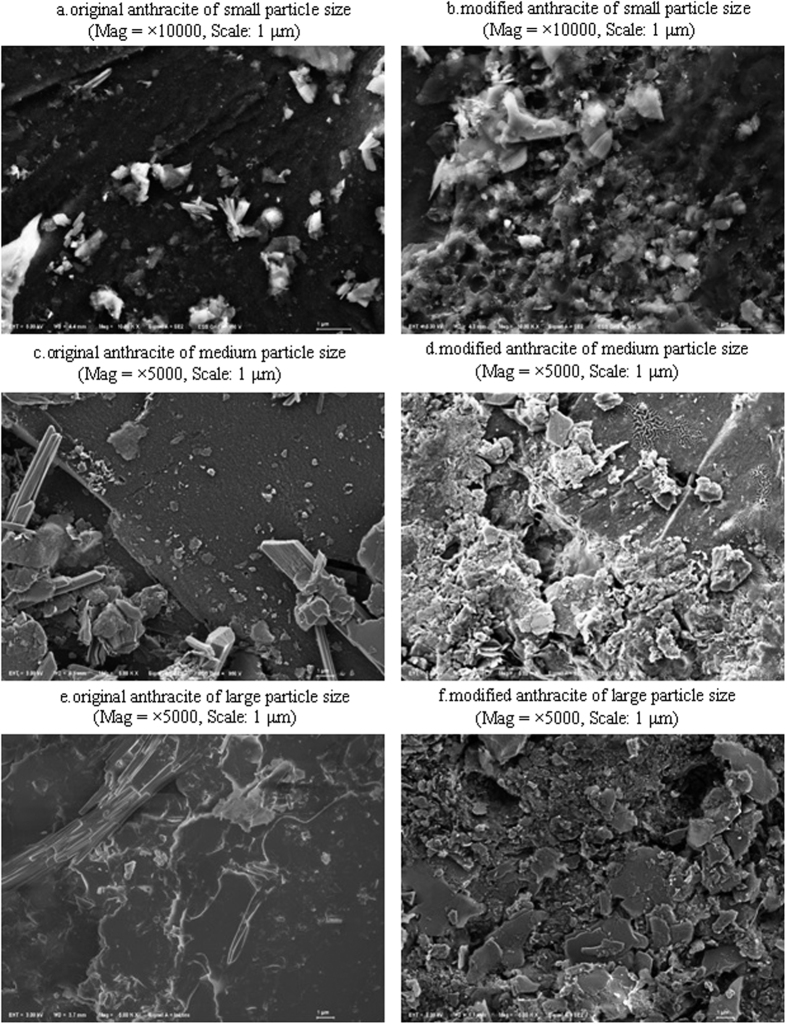
FESEM images of the original and modified anthracite of different particle sizes, with MgAl-LDHs coated anthracite in column No. 9 as an example.

The granule scale and coverage of LDHs-coating film based on those FESEM images were calculated using grid method. The size of micro unit of the grid superimposed on the image was 25*25 mm, which is 0.66*0.66 μm according to the scale. The deviation of the calculation on average coverage was 2.5%. As for the MgAl-LDHs coated anthracite shown in Fig. 1, the average coverage of LDHs-coating film was 60%, 65% and 45%, with the average scale of 4.8 × 8.0, 6.7 × 18.3 and 5.8 × 13.3 (μm, width × length), for the small, medium and large particle sizes, respectively. Compared to the smooth surface of the original anthracite, the surface of modified substrates were covered with white nanocrystallites with different scales and densities, which became fractured with compact porous structure for the medium and large particles, but were denser and more blurring for the small particles. However, the coarse surface starting with LDHs turned to be smooth after the experiment. That might be attributed by the formation of microbial biofilms naturally, as no sterilization procedure was performed during the experiment. The effect of LDHs-coatings on substrate microbial biomass and community dynamics needs further study in the next step.

### Average removal efficiencies of LDHs-modified substrates

The average removal efficiencies of COD, NH_4_^+^-N and TN for all the tested substrates ranged from 71.3% to 89.3%, 68.0% to 91.6% and 53.7% to 89.8%, respectively. NO_3_^−^-N concentrations increased in most columns, with the average accumulation rates ranging from 78.4% to 595.5% (Table 1). The average RSD (relative standard deviation) values for each particle size were all below 15% for the removal efficiencies of COD and NH_4_^+^-N, with particularly smaller variation (around 6%) for the medium particle size, and ranged from 17% to 25% for that of TN, largely due to the great fluctuations of NO_3_^−^-N in the columns. The small RSD values showed that the removal efficiencies of COD and NH_4_^+^-N remained stable for the small-scale column systems for wastewater purification, but the removal of NO_3_^−^-N was relatively poor.

There are limited research studies on the LDHs-modified anthracite for nitrogen removal. In available reports, the original anthracite has been shown to have good performance on phosphorus removal, but their removal performance for TN and NH_4_^+^-N was poor, just around 22% and 28% with the granule size of 8–12 mm^10^. The improvement of 10–20% on nitrogen removal would be good enough in wastewater treatment. In our experiment, the medium anthracite particles with MgCo-LDHs coating achieved high nitrogen removal rates up to 86%, with the removal rates of TN and NH_4_^+^-N increased by 28% and 12%, respectively. It suggested that LDHs-coating modification was promising to improve the comprehensive abilities of anthracite for both nitrogen and phosphorus removal from wastewater.

### Effect of particle size on COD removal

**Figure 2:**
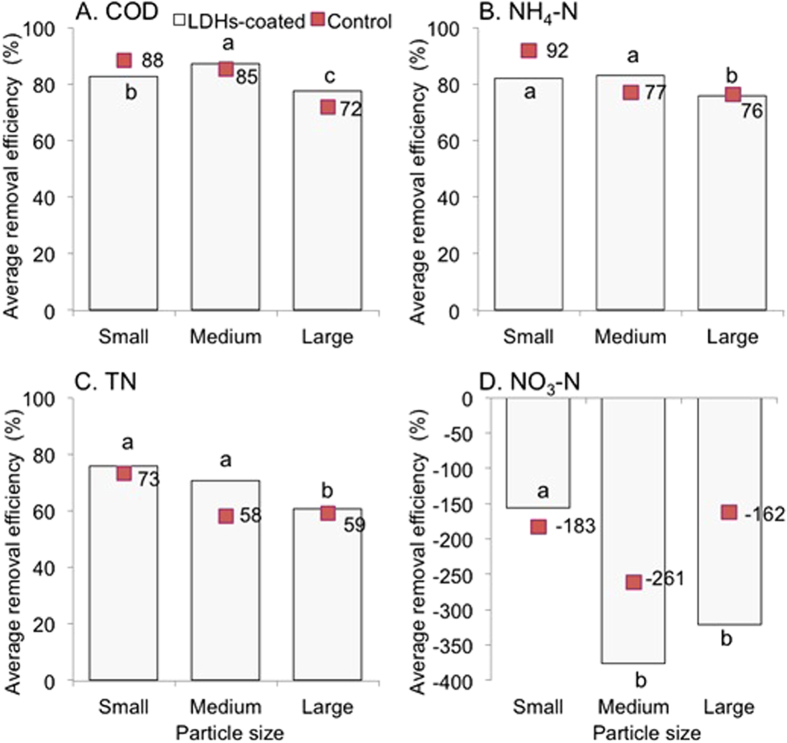
Average removal efficiency (%) of ammonia (NH_4_-N), total nitrogen (TN), nitrate (NO_3_-N) and COD for the LDHs-coated and control substrates of different particle sizes. Different letters above the bar indicated significant difference at *P* ≤ 0.05 according to two-way ANOVA.

Particle size significantly influenced the treatment performance of the modified anthracites (*P* < 0.001), according to the two-way ANOVA (Fig. 2). Medium particle size (1–3 mm) for the modified anthracites was the best for COD removal, with the average removal efficiency all above 85%. COD removal by the small particle size (0.5–1 mm) was slightly lower and no better compared to the control, which suggested that the formation of a dense film on the small particles might not conducive to the growth of aerobic microorganisms^26^,^28^, though small particles had a larger surface area. By contrast, the large particles (3–5 mm) did not have a surface area large enough to support ample microorganisms and surface adsorption^8^,^25^,^29^, so their performance was poor. However, COD removal achieved 99–119%, 100–105% and 86–96% of the control for the large, medium and small particle sizes, respectively. This indicated that the enhancement effect of LDHs-coatings on COD removal was more apparent, or easier to realize, for the larger size of particles. This finding could be largely due to the greater morphological changes after LDHs-coating modification (Fig. 1).

### Effect of particle size on nitrogen removal

**Figure 3:**
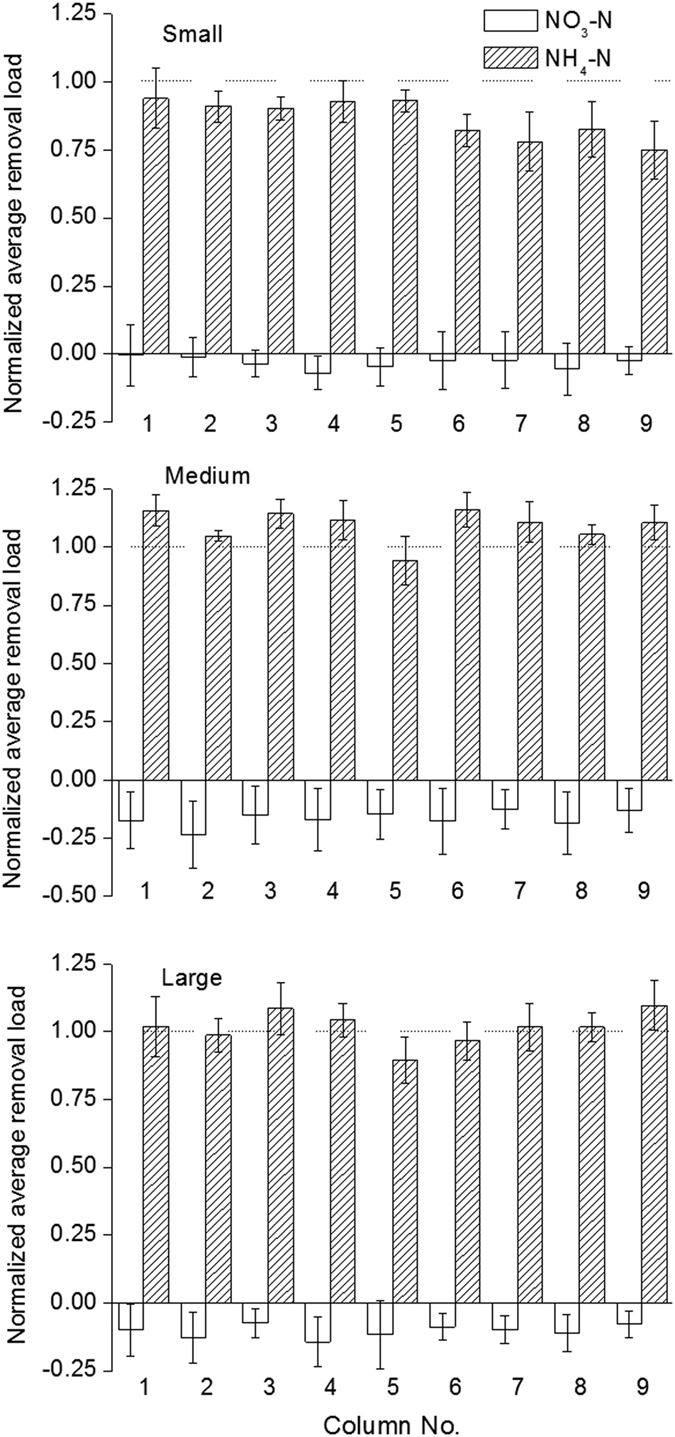
Normalized average removal load (each divided by the ammonia removal load of the control column) of ammonia (NH_4_-N) and nitrate (NO_3_-N) for the modified substrates of different particle sizes (Error bars represent the standard deviations).

Removal efficiencies of different forms of nitrogen significantly varied among different particle sizes (*P* < 0.001) (Fig. 2). In general, the modified anthracites of small and medium sizes achieved better removal of NH_4_^+^-N and TN compared to the large size. The result was in good accordance that the small and medium sizes with the larger BET showed better treatment performance than the large one after LDHs modification. However, the small one was most efficient for removing NO_3_^−^-N. This important evidence was confirmed by quantification of the removal loads for the main nitrogen species, i.e., NH_4_^+^-N and NO_3_^−^-N, in the simulated vertical-flow constructed wetland columns (Fig. 3). It was clearly shown that NH_4_^+^-N has been removed adequately, while a certain proportion of NO_3_^−^-N accumulated, but far less than that of NH_4_^+^-N removed. The accumulation of nitrate varied among different particle sizes in the order of Medium > Large ≫ Small. Ammonia oxidation is the predominating process for the latter two, both of which resulted in considerable nitrate accumulation. However, nitrate accumulation was moderate for the small particle size, suggesting conventional nitrification-denitrification occurred simultaneously. It may be explained that due to low oxygen diffusion in substrates with a small particle size, denitrification was favored under such anoxic or anaerobic condition. Meanwhile, aerobic metabolism might thus be partially inhibited, which in turn explained relatively poor COD removal with the small modified anthracites, because organic matters were mainly degraded by aerobic heterotrophic bacteria in wastewater treatment systems^29^.

### Effect of LDHs-coating on nitrogen removal

The enhancement effect of the nine modified anthracites also varied, and their effect was significant only for NH_4_^+^-N (*P* = 0.029) and TN (*P* = 0.049). According to the two-way ANOVA, there was significant interaction between LDHs-coating (or metal component) and particle size only for the removal of NH_4_^+^-N (*P* < 0.001). Different particle size affected the enhanced effects of the combination of metals. It is worthy of paying attention that the role of magnesium (Mg) differed between the small group and groups of medium and large sizes in affecting NH_4_^+^-N removal (Table 1). The metal combinations with Mg always showed better performance for NH_4_^+^-N removal compared to that with Zinc (Zn) for the medium and large particles, but the opposite trend was observed for the small particles. This result suggested that the interaction between those trace metal ions and particle sizes could strongly influence the treatment performance, by altering the functional microbial community involved in nitrogen removal. Further evidence on the porosity of the modified substrates, as well as the dynamics of ammonia transformers should be obtained for better understanding of the underline mechanism.

### Comprehensive evaluation of treatment performance

**Figure 4:**
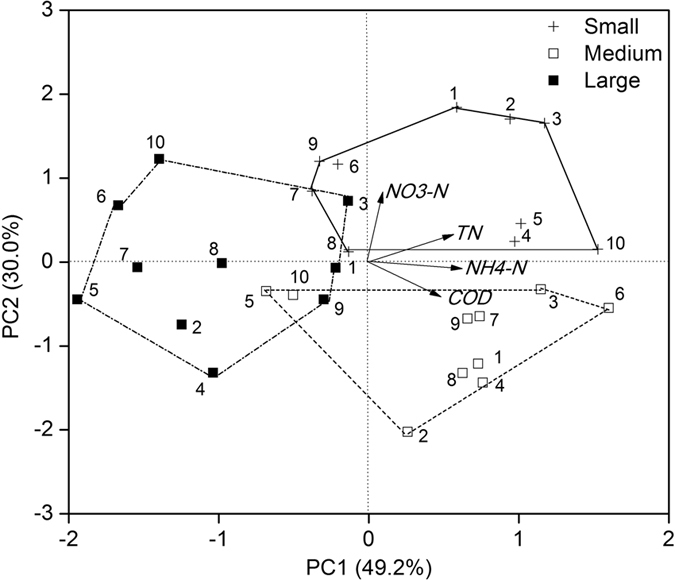
PCA biplot for comprehensive evaluation on the treatment efficiencies of all the tested substrates. Samples are labelled as No. 1–10, which corresponds to the column number.

PCA was used to comprehensively and visually compare the treatment performance of modified anthracites with different particle sizes and LDHs-coatings. In the PCA analysis, principal components (PCs) 1 and 2 explained 49.2% and 30.0% of the total variations, respectively (Fig. 4). The data matrix for PCA analysis composed of the average removal efficiencies of the four parameters for all the 30 tested substrates. Columns filled with the same size of anthracites clearly cluster together, showing comparable performance within each particle size. The divergence between modified anthracites of large size and those of small and medium sizes was shown along PC1, which was mainly contributed by NH_4_^+^-N (40.5%), TN (31.9%) and COD (26.6%), while the difference between the latter two was further revealed along PC2, which was mainly contributed by NO_3_^−^-N (69.2%).

**Figure 5:**
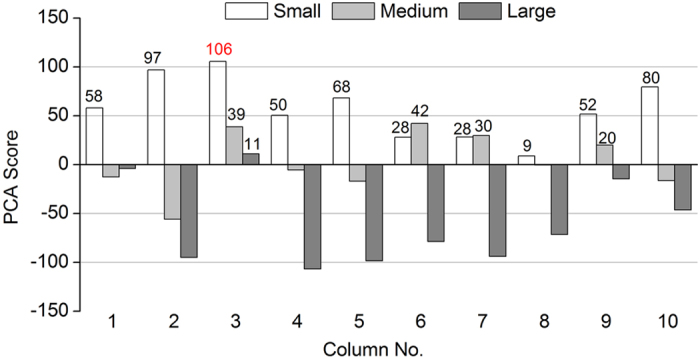
Comprehensive evaluation of PCA scores for all the tested substrates. Positive scores are labeled above the corresponding columns, with the highest score marked in red.

Since the differences of modified anthracites with different LDHs-coatings were still not so evident, the comprehensive PCA scores for each sample were calculated as shown in Fig. 5. It was thus clearly to find that the highest PCA score was obtained by column No. 3 (MgFe-LDHs) with a small particle size. Moreover, column No. 3 got the second place for the medium size groups, next to column No.6 (MgCo-LDHs) (39 vs. 42) and the first place for the large size groups. The result suggested that the modified anthracites coating with MgFe-LDHs could be the best choice of the wetland substrate for wastewater treatment. The conclusion was also supported with related experimental studies on pure LDHs. The mechanisms for efficient removal of pollutants by pure LDHs were mainly through physical-chemical processes, such as anion exchange, adsorption, reconstruction, and weathering/precipitation^30^. Different combinations of the LDH precursor metals could significantly influence the adsorption process. For example, calcined MgAl-LDHs was found the most effective adsorbent of phosphate, followed by MgFe-LDHs, among eight kinds of LDHs^31^. The result suggested a strong interaction between the skeletal Mg and HPO_4_^2−^ ions. As for nitrate removal, the selectivity of MgAl-LDHs for NO_3_^−^ was much lower than those of the other LDHs with Fe, i.e., Mg-Fe, Ni-Fe and Co-Fe^32^. Additionally, in their study, FESEM observation showed that the stacked layer structure was more pronounced after NO_3_^−^ exchange in MgFe-LDHs, compared to the changes in Mg-Al. On the other hand, COD and nitrogen removal was more involved with microbial processes in substrate, which was less studied for pure LDHs materials. It has been demonstrated that the presence of trace metal elements could stimulate or enhance the microbial biomass and activity during sewage treatment^33^,^34^. In particular, the cells of nitrifying bacteria often had a complex structure of the membrane, while small amount of Fe^3+^ or Mn^2+^ ions could help to increase the permeability of the cell membrane, thus speeding up nitrification and the adsorption of nutrients^35^. The detailed mechanism concerning the effect of different kinds of LDHs on enhancing microbial removal processes will be carried out in the next stage.

### Recommendations and perspectives

Significant effect of particle size on nitrogen removal suggested that adsorption was one of the key removal processes, which largely depended on the ion exchange properties of LDHs, regeneration of the LDHs-modified anthracites is thus important and should be well considered for practical engineering application. It has been demonstrated that different divalent metal bases could significantly affect desorption and regeneration performance of LDHs^14^,^36^, and Mg-based LDHs were suggested potentially more applicable for regeneration and reuse than Ca-based LDHs^14^. Further studies are needed to screen out the most efficient LDHs for removing organics and nitrogen while taking their regeneration and reusability, as well as cost efficiency, into account for engineering practices.

## Conclusions

In this study, it is the attempt to apply simple LDHs-modification on substrates to improve the removal efficiency of constructed wetland for sewage wastewater treatment. The study demonstrated LDHs coating as one of the promising strategies to make a breakthrough in the modification of wetland substrates. Results showed that the medium particle size (1–3 mm) was the most effective for LDHs modification which effectively enhanced the removal of nitrogen and organics, while MgFe-LDHs was the best of nine modified coatings tested for anthracite. The long-term performance of the LDHs-modified substrates and their potential in the small-scale planted constructed wetland systems should be evaluated in future, as well as the interaction between the modified substrates and pollutants.

## Additional Information

**How to cite this article**: Zhang, X. L. *et al*. Removal of oxygen demand and nitrogen using different particle-sizes of anthracite coated with nine kinds of LDHs for wastewater treatment. *Sci. Rep*. **5**, 15146; doi: 10.1038/srep15146 (2015).

## Figures and Tables

**Table 1:** Removal efficiencies (%) of COD, ammonia (NH_4_-N) and total nitrogen (TN) and nitrate (NO_3_-N) accumulation rate (%) in all the tested columns.

Columns			COD		NH_4_−N		TN		NO_3_−N	
			Mean	S.D.	Mean	S.D.	Mean	S.D.	Mean	S.D.
Small (n = 8)										
1	Fe	Ca	**75.8**	10.8	87.8ab*	8.5	77.0	10.5	84.2	126.7
2		Zn	81.4	10.7	83.7abc	9.0	83.1	11.4	**78.4**	109.6
3		Mg	81.8	10.2	82.8abc	9.2	**89.8**	19.9	121.9	148.9
4	Co	Ca	84.7	9.3	86.5abc	9.9	75.8	12.2	239.1	239.2
5		Zn	85.9	5.1	85.3abc	9.5	76.3	11.4	191.9	211.7
6		Mg	78.9	8.2	76.4bc	11.1	74.7	13.3	171.0	222.4
7	Al	Ca	82.3	9.4	74.0bc	11.7	69.3	14.3	155.3	226.1
8		Zn	83.6	8.0	78.1abc	14.2	**66.8**	12.8	**240.1**	300.2
9		Mg	83.2	9.1	**71.8c**	10.3	72.1	10.6	102.9	138.9
10	Control		**88.3**	7.2	**91.6a**	9.0	73.2	15.3	182.5	262.9
Medium (n = 5)										
1	Fe	Ca	86.4	7.8	88.2ab	3.2	66.9	10.8	413.6	329.8
2		Zn	86.5	7.9	80.5abc	5.5	71.6	15.9	**595.5**	538.5
3		Mg	86.1	4.9	87.4ab	3.1	77.1	17.5	325.0	309.5
4	Co	Ca	88.9	4.0	85.3ab	5.9	68.9	11.5	437.1	417.8
5		Zn	85.2	9.0	**73.0c**	6.8	61.0	17.0	277.6	203.4
6		Mg	85.8	3.6	**88.8a**	4.6	**86.7**	12.2	419.4	450.0
7	Al	Ca	**89.3**	5.1	84.9ab	4.0	66.5	26.6	282.4	234.8
8		Zn	89.2	3.3	81.6abc	7.1	71.7	10.6	437.3	452.0
9		Mg	88.1	2.3	84.5ab	5.6	67.6	18.3	310.2	266.5
10	Control		**85.2**	5.2	76.9bc	6.5	**58.2**	16.8	**261.2**	205.8
Large (n = 5)										
1	Fe	Ca	83.1	7.8	77.1ab	3.0	68.0	11.5	289.1	283.5
2		Zn	77.2	8.8	73.5bc	2.0	62.4	12.7	458.1	471.6
3		Mg	79.5	5.6	80.7a	2.6	**68.2**	8.3	192.2	111.3
4	Co	Ca	79	9.6	77.7ab	2.3	57.6	11.5	**497.4**	384.5
5		Zn	77.8	12.0	**68.0c**	6.4	53.8	28.1	360.8	359.7
6		Mg	**71.3**	15.9	72.3bc	4.4	62.1	18.8	291.0	225.2
7	Al	Ca	74.2	6.2	75.8ab	3.5	54.9	19.9	331.7	261.1
8		Zn	74.4	8.0	76.2ab	2.4	67.5	11.3	392.4	319.3
9		Mg	**85.5**	7.3	**81.7a**	2.2	**53.7**	12.6	231.0	159.3
10	Control		71.9	17.0	76.2ab	1.5	59.0	11.7	**162.1**	100.5

^*^Different letters in the postfix indicated that they were significantly different at *P* ≤ 0.05; maximum and minimum of the same treatment group were underlined in bold, respectively.
